# Individual Variability of *Nosema ceranae* Infections in *Apis mellifera* Colonies 

**DOI:** 10.3390/insects3041143

**Published:** 2012-11-01

**Authors:** Grace E. Mulholland, Brenna E. Traver, Nels G. Johnson, Richard D. Fell

**Affiliations:** 1 Department of Entomology, Virginia Tech, 324 Price Hall, Blacksburg, VA 24061, USA; E-Mails: gracem@vt.edu (G.E.M.); rfell@vt.edu (R.D.F.); 2 Laboratory for Interdisciplinary Statistical Analysis (LISA), Department of Statistics, Virginia Tech, 405 Hutcheson Hall, Blacksburg, VA 24061, USA; E-Mail: nels@vt.edu

**Keywords:** *Nosema ceranae*, *Nosema apis*, *Apis mellifera*, real-time PCR, spore count, variability

## Abstract

Since 2006, beekeepers have reported increased losses of *Apis mellifera* colonies, and one factor that has been potentially implicated in these losses is the microsporidian *Nosema ceranae*. Since *N. ceranae* is a fairly recently discovered parasite, there is little knowledge of the variation in infection levels among individual workers within a colony. In this study we examined the levels of infection in individual bees from five colonies over three seasons using both spore counting and quantitative real-time PCR. The results show considerable intra-colony variation in infection intensity among individual workers with a higher percentage of low-level infections detected by PCR than by spore counting. Colonies generally had the highest percentage of infected bees in early summer (June) and the lowest levels in the fall (September). *Nosema apis* was detected in only 16/705 bees (2.3%) and always as a low-level co-infection with *N. ceranae*. The results also indicate that intra-colony variation in infection levels could influence the accuracy of *Nosema* diagnosis.

## 1. Introduction

Disease conditions with the symptoms of nosemosis were a concern for beekeepers even before the discovery and classification of *Nosema apis* as a microsporidian by Zander in 1909 [1]. Microsporidia are obligate intracellular parasites of both vertebrates and invertebrates, and numerous studies have been conducted on the impacts of *N. apis *and its pathological effects on honey bees since its initial identification [1,2,3]. Almost 90 years later, a second *Nosema* species, *N. ceranae*, was described from the Asian honey bee, *Apis cerana* [4] and then later in the European honey bee *Apis mellifera* [5,6,7]. Nosema disease caused by these microsporidia occurs worldwide in honey bees and has been shown to have a number of negative impacts on honey bee colonies, depending on the geographic location and the *Nosema* species involved. Damage to colonies may entail shortened worker lifespans [8], reduced colony build-up and productivity [9], queen supersedure [1,10,11], increased winter losses [12], and colony collapse [13,14]. 

The importance of *Nosema* as a potentially serious pest of honey bees has been recognized since its discovery, but the disease has not always been given sufficient consideration in management practices. This lack of attention may be due partly to variation in disease prevalence in different areas of the world as well as seasonal variation in infection intensity. In temperate areas, disease prevalence has been shown to vary among regions [15,16,17] and years [11], and among apiaries and colonies within apiaries [18,19]. Infection intensity has also been shown to vary considerably during the course of a year, as well as between years for both *N. apis* [19,20,21] and *N. ceranae* [22,23]. In temperate regions, *N. apis* infections typically peak in the spring, decrease during the summer and then increase again in the fall before declining during the early winter months [8,20,21,24]. *N. ceranae*, on the other hand, can be detected in all four seasons [25] and may show less seasonality. Martin-Hernández *et al.* [22], for example, reported a loss in seasonality of *Nosema* diagnosis over a seven-year period, such that by 2005 no seasonal differences were found in the percent of positive samples during the year. Traver *et al.* [23], however, reported seasonal differences in the levels of infection over a 13-month sampling period. Infection levels peaked in late spring (April, May and June) and then declined during the summer. Infection levels remained low during the fall and winter and did not increase significantly until April. This cycle was similar to that observed in Germany [26]. Copley and Jabaji [27] also found similar patterns in infection levels in the intestines of bees analyzed during the warmer months; infection levels peaked in late spring and then declined during the summer. 

The variable nature of *Nosema* infections complicates management practices particularly with regard to treatment decisions. These problems may be further complicated by difficulties in sampling, sample interpretation, and the *Nosema *species involved [28,29]. The standard procedure for *Nosema* sampling involves the use of composite samples and spore counting to determine average spore numbers per bee [30,31]. Sample size recommendations vary from a minimum of 10–25 bees [20,30] to 60 bees [31], to as many as several hundred, depending on the level of infection and the desired probability of detection [1]. Most studies also recommend sampling foragers at the hive entrance, as it is thought that foragers tend to have higher levels of infection [21,29,32]. In colonies infected with *N. ceranae*, Higes *et al.* [13] found that the proportion of foragers infected was the only useful indicator of the level of disease in a colony. In contrast, Traver *et al.* [23] examined in-hive bees as opposed to foragers for *Nosema* to provide an estimate of colony infection levels, since foragers are not present during the winter months and only represent about 25% of a colony’s population [33] during the warmer months. They used both real-time PCR and spore counts and found no significant differences in the levels of infection for bees sampled from the brood nest, the fringe of the brood nest, or from the supers during any month. They also found no significant differences in the levels of infection of workers sampled from either the inner or outer edges of the winter cluster [23]. Because these studies were based on composite samples, however, no information was provided on the proportion of bees infected with either *N. ceranae* or *N. apis*. Several authors have argued that the examination of individual bees is a more meaningful indicator of colony infection levels than the use of average spore numbers from a composite sample [13,19,21,34]. Meana *et al.* [29] found that spore counts varied greatly between house and forager bees, and among foragers sampled on different days, indicating that spore counts were not a useful measure of colony health. A figure based on the proportion of bees in a colony infected with *Nosema* may serve as a more reliable indicator of colony health [13,21,29]. However, the degree of infection within individual bees is also important [19] and needs to be considered before management decisions are made.

In this study, we examined the level of *N. ceranae* and *N. apis* infections in individual bees using both spore counts and real-time PCR (qPCR) during the spring, summer and fall seasons. Our goal was to gain a better understanding of the variation in infection levels among individual bees within a colony and to determine how this variation might affect sampling, results, and interpretation. We were particularly interested in addressing this question uniquely through a combination of spore counting and qPCR analysis which has not been done before. 

## 2. Results and Discussion

A total of 705 individual bees were analyzed from five colonies over three seasons in 2010. Colonies used were part of a previous year-long monitoring study. These five colonies had been found naturally infected with *N. ceranae* over a 13-month period, and data from this study match the seasonal trends previously observed [23]. Furthermore, bees analyzed were sampled from the honey supers because we found no significant difference in infection levels among different groups of bees sampled [23]. We also used in-hive bees instead of foragers to gain a better understanding of how variable infections are inside the colony, as foragers only represent about a quarter of the colony population [33]. 

**Figure 1:**
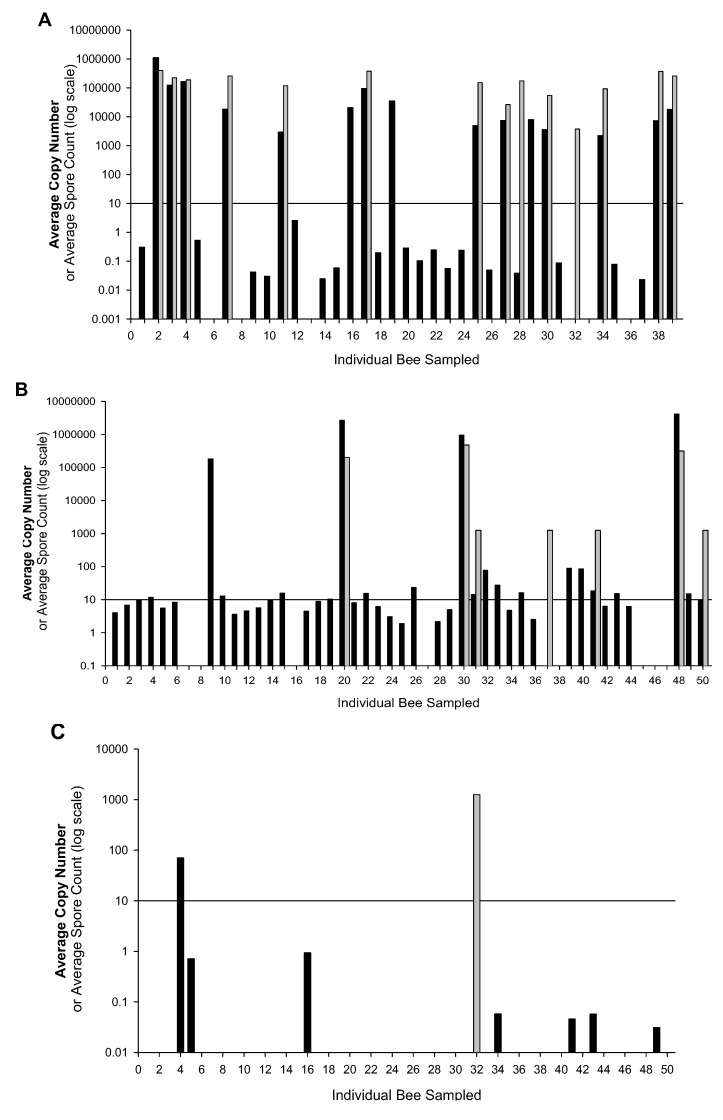
Variation in the mean number of *N. ceranae* DNA copies (in a standardized 50 ng sample/bee) and spore counts for the same individual bees. The line represents a cut-off point, 10 copies, at which we can reliably detect *N. ceranae* levels. Black bars represent the mean *N. ceranae* DNA copies from each bee sample while the gray bars represent the mean spore counts for the same bee. **(a)** Data from colony 3 in the spring **(b)** Data from colony 1 in the summer **(c) **Data from colony 3 in the fall.

Overall, colonies were found to be infected with *N. ceranae* (Table 1). In the spring, all colonies (5/5) were found infected with an average *N. ceranae* copy number of 8,007 ± 5,301 (n = 212). In the summer, 80% of colonies (4/5) were found infected with an average *N. ceranae* copy number of 37,057 ± 20,097 (n = 243). Only one colony (1/5) was found infected in the fall; however, only one bee from this colony was positive for *N. ceranae* infection with an average copy number of 70.3. All other samples were negative (n = 249). When *N. ceranae* was detected, we analyzed infection levels for all samples with 10 or more copies. We rated samples with fewer than 10 copies of *N. ceranae *as positive for infection, but we did not include these values in estimating infection levels. Adjusted average copy numbers for samples with values over 10 copies (threshold qPCR) are shown in Table 1, while all data are shown in Figure 1 with a line indicating the 10-copy cut-off value. 

**Table 1:** Real-time PCR data for colonies over three seasons in 2010. Copy numbers reflect mean *N*. *ceranae* DNA copies in a standardized 50 ng DNA extract from individual worker bees.

	Spring		Summer		Fall	
Colony	Avg. Copy # ^1^	N ^2^	Avg. Copy #	N	Avg. Copy #	N
1	1099.66 ± 1099.66	1/43	159871.87 ± 99440.99	19/50	0 ± 0	0/50
2	0.61 ± 0.61	1/50	6.52 ± 1.62	16/50	0 ± 0	0/50
3	42400.72 ± 28832.82	15/39	6.05 ± 1.67	13/50	1.41 ± 1.41	1/50
4	250.45 ± 250.45	1/44	0 ± 0	0/43	0 ± 0	0/50
5	773.69 ± 450.84	3/36	20212.60 ± 14470.56	6/50	0 ± 0	0/50

^1^ Average copy number ± the standard error of the mean (SEM). The average copy number has been adjusted so that only data greater than 10 copies are shown (threshold qPCR). The average copy number may be less than 10 copies when only a few bees from each season were infected;^ 2^ N represents the number positive by qPCR over the total number of bees sampled from each colony.

Spore counts ranged from zero to 453,750 in the spring with an average of 18,538 ± 4,891 and a median of zero. In the summer, spore counts were the highest with a range from zero to 3,962,500 with an average of 34,717 ± 20,560 and a median of zero. Spore counts were lowest in the fall with a range of zero to 1,250 and a median of zero. Spore analyses for individual colonies are reported in Table 2. 

**Table 2:** Spore analysis for colonies over three seasons in 2010.

	Spring		Summer		Fall	
Colony	Avg. # Spores ^1^	N ^2^	Avg. # Spores	N	Avg. # Spores	N
1	5988 ± 5929	3/43	19950 ± 11919	7/50	0 ± 0	0/50
2	50 ± 35	2/50	75 ± 55	2/50	0 ± 0	0/50
3	68622 ± 19132	14/39	50 ± 35	2/50	25 ± 25	1/50
4	85 ± 63	2/44	145 ± 62	5/43	25 ± 25	1/50
5	27500 ± 16044	3/36	148525 ± 98311	7/50	50 ± 35	2/50

^1^ Average spore count ± the standard error of the mean (SEM);^ 2^ N represents the number positive from spore analysis over the total number of bees sampled from each colony.

When we examined the ability of either method to detect *Nosema* infections, the type III tests of fixed effects of season by method interaction (*i.e.*, spore or qPCR analysis) was significant (Num DF = 2; Den DF = 12; F = 5.31; p = 0.02). Therefore we examined the difference between the methods by season and found that there was a significant difference in the log odds for the summer season only (DF = 12; t-stat = 3.8; p = 0.0025). In the spring as *N. ceranae* levels were increasing, spore and adjusted qPCR analysis were identical in their ability to detect *Nosema* infections (Table 3; DF = 12; t-stat = −0.51; p = 0.62) and equal in their ability to detect the absence of *N. ceranae* infections in the fall (Table 3; DF = 12; t-stat = −1.25; p = 0.24). In the summer, more infections were observed using qPCR *versus* spore analysis (Table 3). 

*Nosema apis *was detected by qPCR in only 16 (2.3%) of the total bees (n = 705) examined. *N. apis* was at its highest level in the spring with an average copy number of 32.7 ± 9.3 for the three colonies where it was observed (colony 3, in 28.2% of the bees examined; colony 4, in 2.3%; and colony 5, in 8.3%). *N. apis *was only detected in one bee during the summer (2% of colony 5) with an average copy number of 27.3 ± 27.3. *N. apis* was absent from all bees sampled during the fall season. Every *N. apis* infection occurred as a co-infection with *N. ceranae*, with high levels of *N. ceranae* and low levels of *N. apis* as previously observed [15]. This finding provides support for the suggestion by Paxton *et al.* [35] that *N. ceranae* is replacing *N. apis* in *A. mellifera*. 

qPCR and spore counts for *N. ceranae* on the 705 bees sampled were assessed for correlation. The weak correlation (r = 0.22, n = 705) between the number of *Nosema *spores and the average number of *N. ceranae *copies in the individual bees supports the observation that *N. ceranae* is not a heavy producer of spores, unlike *N. apis*. Spores are thus not a strong indicator of the total *N. ceranae* infection level in honey bee midgut cells [28]. High spore counts are also not always indicative of unhealthy colonies [29] as mean spore counts will vary throughout the year and are not always directly related to the parasite level [13]. qPCR appears to be more effective at detecting low levels of *N. ceranae*, having detected an equal or greater proportion of infections in individual bees than spore counting in every colony sampled (Table 3 and Figure 1). Spores were detected in 1.6% (11/705) of bees that were negative for infection by qPCR, but all such cases involved the detection of only one spore during spore counting.

**Table 3:** Percentage of bees sampled from each colony determined infected by spore analysis and qPCR over 3 seasons.

	Spring			Summer ^3^			Fall		
Colony	Spore Counts	Raw qPCR ^1^	Threshold qPCR ^2^	Spore Counts	Raw qPCR	Threshold qPCR	Spore Counts	Raw qPCR	Threshold qPCR
1	7	32.6	2.3	14	82	38	0	12	0
2	4	36	2	4	56	32	0	2	0
3	35.9	84.6	38.5	4	56	26	2	14	2
4	4.5	63.6	2.3	11.6	16.3	0	2	10	0
5	3	63.9	8.3	14	18	12	4	12	0

^1^ All samples with a positive result for qPCR are included in this percentage; ^2^ Only samples with a copy number above 10 are included in this percentage; ^3^ Differences in detection between spore analysis and threshold qPCR were significantly different (p < 0.05) in the summer season only.

Figure 1 shows the prevalence of low-level infections in some colonies. We speculate these low-level infections are not problematic but could lead to higher levels in the summer if additional stressors are involved. In all five hives examined in this study, infection levels declined to almost zero by fall [23]. Low-level infections or new infections could weaken bees; epithelial lesions have been observed in the midgut of infected bees and it has been suggested that this could increase the bees’ susceptibility to viral infections [13]. Other factors including poor weather or exposure to pesticides could also trigger an increase in infection levels thereby negatively impacting colony health [36]. 

It is not surprising that *N. ceranae* was detected in all colonies due to the widespread, global prevalence of the pathogen [15,37,38,39,40,41,42,43,44,45,46,47,48,49,50] and the observation that *Nosema* appears to be in most colonies, even healthy ones [15,19]. One must note that although *N. ceranae* is detected, the incidence level is important as is the sampling scheme. Standardized sampling protocols for *Nosema* have not been consistent. Composite sampling [19,30,51,52,53,54] and individual bee sampling [20,24,55,56,57,58] methods have each been used. Any sampling scheme must take into account the number of bees and number of colonies to be sampled as well as the sampling time and analysis costs. Individual bee analysis takes much more time than composite sampling, so composite sampling is more efficient for large sampling schemes. Composite samples, however, provide an estimated average infection level for the colony rather than an estimate of the percentage of bees infected in a colony and the level of infection in those individuals. Furthermore, the variation in infection intensity observed among individuals and the uneven distribution of infection throughout the colonies further confound the accurate assessment of a colony’s infection level [21] and complicate treatment decision making.

Composite sampling of 25 [19,54] and 30 [21,34] bees has been used for diagnosing *Nosema* infection in colonies. Results from composite samples of 25 bees [54] were comparable to examining 10 individual bees as similar mean spore counts were obtained for each method. When 30 bees were analyzed in composite samples, a large range of variation was observed among samples, with many bees negative for spores but showing the early stages of infection in midgut cells [21]. When comparing composite sample analysis to the analysis of individual bees, individual bee analysis was better for determining the presence or absence of *Nosema* in a colony [13,34]. A significant correlation coefficient has been found between the number of infected bees and the average number of spores per bee in composite samples for *N. apis* [2,34,59]. A similar correlation was found between the infection level in a composite sample and the percentage of infected bees in low to moderate level infections [60]. However, in a different study in which 25 individual bees were examined, no correlation between the mean spore count per bee and the percentage of infected bees was found in the sample due to a high number of spores present in a few bees [53]. Composite samples with only one or a few highly infected individuals can present a skewed infection level for a colony [19,53,60,61]. For example, a few heavily infected bees in a composite sample could generate the same spore count as a sample with many moderately infected bees [34]. If no spores are observed, either no infection or a low-level infection that may be detected by qPCR is present, but most likely such infections are not economically significant.

The results from this study demonstrate considerable intra-colony variation in infection intensity among individuals and between analysis techniques. The high infection levels observed in several of the colonies in the spring were detected by both qPCR and spore analysis, but spore analysis may provide a very different picture of the proportion of bees infected when infection levels are low (Figure 1B). This variation is important to note because it illustrates a major concern of sampling and the techniques used for analysis. Generalizations of the infection level of a colony may be inaccurate depending on whether composite or individual bee samples are used and the technique used to assess infection. *N. ceranae *infection levels can vary substantially both within colonies and among seasons. The analysis of composite samples using spore counts could lead to misdiagnosis of a hive (either overestimating or underestimating the overall infection level) depending on which bees are analyzed. These findings also bring into question whether a hive that has been deemed highly infected has been diagnosed as such because of an overall high infection level or because of sampling practices in which a minority of bees were highly infected but the remainder exhibited only low-level infections or no infection. The results of this study point to the need for not only developing standardized sampling techniques for assessing *N. ceranae* infections, but also for a better understanding of how such results should be applied to colony management decisions.

## 3. Experimental Section

### 3.1. Sample Collection

All colonies were located in apiaries owned by Virginia Tech (Blacksburg, VA, USA). Each hive was sampled at the beginning of 3 seasons: spring (March), summer (June), and fall (September) of 2010. Between 36 and 50 bees were collected from each of 5 hives during each season; this sample size was determined to suffice for a 95% probability of detecting disease given the infection rates of the colonies sampled as determined in a previous study [62]. Colonies sampled for this study had been used in a previous study [23] and were known to be infected with *N. ceranae* at a level ≥ 20%. Bees were collected from the honey supers (the top hive bodies in which honey is stored) and stored in 70% ethanol prior to analysis.

### 3.2. Genomic DNA Extraction

In order to extract genomic DNA to be used for qPCR, the abdomen of each bee was homogenized in Bender buffer and subjected to a phenol:chloroform extraction and isopropanol precipitation as previously described [23]. The extracted DNA was resuspended in DEPC-treated water overnight at room temperature and quantified on a Nanodrop2000 Spectrophotometer (Thermo Scientific, Wilmington, DE). Sample extracts were diluted to a standardized 10 ng/µL before analysis.

Following the first centrifugation in the phenol:chloroform DNA extraction, the organic phase was not discarded as per usual protocol but instead saved. The phenol in this remaining portion was solubilized with 95% ethanol (raising the total volume of solution to 1 ml) so that a spore count could be performed for each bee. This modification was performed so that the genomic data for each bee could be compared to the spore count data of the same individual.

### 3.3. Spore Counting

Each abdomen was crushed in a total volume of 1 ml using a 1-ml pestle. The number of spores contained in a sample of each lysate was determined through counts on a Bright-line hemocytometer (Hausser Scientific, Horsham, PA). Counts were performed twice for each lysate and any sample initially deemed to show one spore or fewer was re-counted twice to validate the result. The number of spores in each bee was then calculated as previously described [63].

### 3.4. Quantitative Real-time PCR (qPCR)

A qPCR analysis was performed for each sample using primers and probes designed from 16S SSU rRNA for both *N. ceranae* and *N. apis*, as described previously [64]. Each well in the PCR plate was loaded with 50 ng of DNA, and all samples were run in triplicate and alongside standards for both species. Standard curve quantification was used to convert the resulting cycle threshold (C_T_) values to the number of copies of *N. ceranae *and *N. apis* present in each sample. Adjusted qPCR results were calculated whereby samples with fewer than 10 copies of *Nosema* DNA were conservatively deemed negative for infection due to the questionable sensitivity of the assay close to its level of detection. 

### 3.5. Statistical Analysis

To determine if there was a difference between the ability to detect infection using spore analysis and using qPCR, we used a binomial logistic regression model fit using proc glimmix in SAS 9.2. The model we fit is as follows: where *Y_ijk_* is the number of bees out of the sample of n*_ij_* bees from colony *i* in season *j*, where an infection was detected using method *k*, *μ_0_* is the overall log odds of detecting an infection, *α_i_* is the random intercept for colony *i*, *βj* is the fixed effect for season *j*, *b_ij_* is the specific random effect for season *j* for colony *i*, *τ_k_* is the fixed effect for detection method *k*, and (*βτ*)*_jk_* is the season by detection method interaction for season *j* and treatment *k*. If the test of type III sums of squares for fixed effects was significant, comparisons of least squared means were computed for testing differences between methods of detection within season. Significance was selected as α = 0.05. 


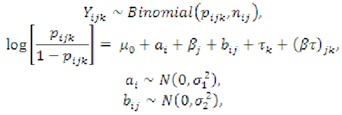


## 4. Conclusions

The examination of individual bees for *Nosema* infections using both spore counts and qPCR showed variation in the percentage of infected bees and intensity of infections within colonies, as well as differences based on analysis techniques. Spore counts and qPCR gave similar percentages of infection and infection intensities for colonies sampled in the spring and fall, but a higher percentage of low-level infections was detected by qPCR in the summer. Most infections were caused by *N. ceranae* with *N. apis* detected in only 2.3% of the samples, all of which were low-level co-infections with *N. ceranae*. Infection levels and the percentage of infected bees declined in all five colonies in the fall. The high degree of variability between individual bee infections and the large number of low-level infections raises questions as to how such results should be applied to colony management decisions. Our data indicate that the use of spore counts would provide acceptable estimates of colony infections for *N. ceranae* in the spring and fall, but not during the summer.
